# Interactions between the HIV-1 Unspliced mRNA and Host mRNA Decay Machineries

**DOI:** 10.3390/v8110320

**Published:** 2016-11-23

**Authors:** Daniela Toro-Ascuy, Bárbara Rojas-Araya, Fernando Valiente-Echeverría, Ricardo Soto-Rifo

**Affiliations:** Molecular and Cellular Virology Laboratory, Virology Program, Institute of Biomedical Sciences, Faculty of Medicine, Universidad of Chile, Independencia 834100, Santiago, Chile; barbara.rojas.araya@gmail.com (B.R.-A.); fvaliente@med.uchile.cl (F.V.-E.)

**Keywords:** HIV-1 unspliced mRNA, mRNA decay, REV, UPF1, Staufen, m^6^A, YTHDF2

## Abstract

The human immunodeficiency virus type-1 (HIV-1) unspliced transcript is used both as mRNA for the synthesis of structural proteins and as the packaged genome. Given the presence of retained introns and instability AU-rich sequences, this viral transcript is normally retained and degraded in the nucleus of host cells unless the viral protein REV is present. As such, the stability of the HIV-1 unspliced mRNA must be particularly controlled in the nucleus and the cytoplasm in order to ensure proper levels of this viral mRNA for translation and viral particle formation. During its journey, the HIV-1 unspliced mRNA assembles into highly specific messenger ribonucleoproteins (mRNPs) containing many different host proteins, amongst which are well-known regulators of cytoplasmic mRNA decay pathways such as up-frameshift suppressor 1 homolog (UPF1), Staufen double-stranded RNA binding protein 1/2 (STAU1/2), or components of miRNA-induced silencing complex (miRISC) and processing bodies (PBs). More recently, the HIV-1 unspliced mRNA was shown to contain *N*^6^-methyladenosine (m^6^A), allowing the recruitment of YTH *N*^6^-methyladenosine RNA binding protein 2 (YTHDF2), an m^6^A reader host protein involved in mRNA decay. Interestingly, these host proteins involved in mRNA decay were shown to play positive roles in viral gene expression and viral particle assembly, suggesting that HIV-1 interacts with mRNA decay components to successfully accomplish viral replication. This review summarizes the state of the art in terms of the interactions between HIV-1 unspliced mRNA and components of different host mRNA decay machineries.

## 1. Introduction

Eukaryotic cells employ quality control mechanisms to ensure that each step of mRNA metabolism, from transcription to translation and decay, is properly executed in space and time and the genetic code is correctly expressed. mRNA surveillance and decay pathways are responsible for recognizing aberrant mRNAs that arise due to errors in the DNA template or by misprocesses occurring during mRNA biogenesis [1]. As such, efficient and accurate gene expression is ensured by mechanisms that degrade mRNAs in the nucleus as a response to defects in transcription elongation [2], splicing [3], 3′-end formation [3], and nuclear export [4]. Following export to the cytoplasm, nonsense-mediated decay (NMD), a ribosome-coupled quality control mechanism, induces degradation of mRNAs that contain premature termination codons [5]. Other translation-dependent mechanisms of mRNA degradation are the no-go decay (NGD) pathway, which leads to endonucleolytic cleavage of mRNAs containing strong stalls in translation elongation [6,7], and the non-stop decay (NSD), which corresponds to a quality control mechanism that detects mRNA molecules lacking a stop codon [8]. In addition, some mRNAs harbor specific *cis* signals including miRNA target sites, AU-rich elements, and methylated adenosines (*N*^6^-methyladenosine or m^6^A), which have been involved in the control of mRNA stability [9,10,11,12,13,14].

Upon viral infection, host cells mount an antiviral stress response in order to create a hostile environment for viral replication. This cellular response usually involves the shut-off of protein synthesis and the concomitant assembly of RNA granules such as stress granules (SGs), which correspond to sites of mRNA triage and PBs, which contain the mRNA degradation machinery [15]. Given the fact that most positive single-stranded RNA viruses including poliovirus (PV), hepatitis C virus (HCV), and human immunodeficiency virus (HIV) use the same molecule first as mRNA and then as the packaged genome, it is not surprising that these viruses have evolved different mechanisms aimed at modulating the assembly of different RNA granules and counteracting mRNA decay machineries [15]. Indeed, there is increasing evidence indicating that these viruses are able to interact with and/or modify the cellular factors implicated in mRNA quality control mechanisms during different steps of their replication cycle [15].

This review summarizes the state-of-the-art in terms of the interactions between the HIV-1 unspliced mRNA and proteins with cellular factors involved in different mRNA decay pathways. We also discuss the potential strategies the virus has evolved to divert some of these mRNA degradation pathways or their components and to favor viral gene expression and replication.

## 2. An Overview on Human Immunodeficiency Virus Type-1 (HIV-1) Gene Expression

**Figure 1:**
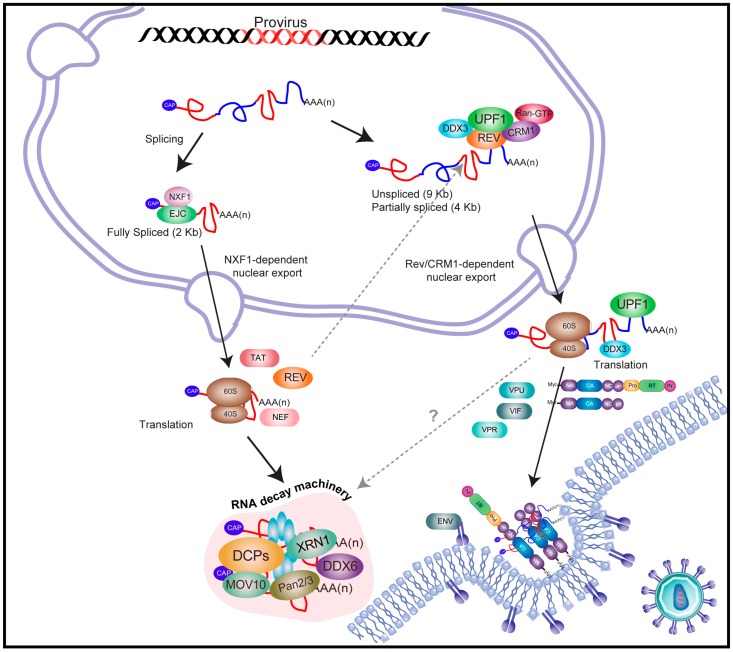
Post-transcriptional control of gene expression in HIV-1. Upon RNA polymerase II-driven transcription, the capped and polyadenylated 9 kb full-length mRNA undergoes alternative splicing in order to generate the 2 kb fully spliced and the 4 kb partially spliced (omitted for simplicity) transcripts. Fully spliced transcripts follow the canonical pathway for mRNA metabolism, in which nuclear export and translation are ensured by the splicing-dependent recruitment of nuclear factors such as the exon junction complex (EJC) and the mRNA nuclear export factor NXF1. Once in the cytoplasm, fully spliced mRNA recruits the host translational machinery in order to synthesize viral proteins TAT, REV, and NEF and upon several rounds of translation they are degraded by the host RNA decay machinery. The viral protein REV enters the nucleus, allowing the accumulation of the 9 kb unspliced mRNA and its subsequent nuclear export through the chromosomal maintenance 1 (CRM1)-dependent pathway. This alternative nuclear export pathway allows the unspliced mRNA to evade surveillance and quality control mechanisms associated with the canonical nuclear export pathway. During its journey to the cytoplasm, the unspliced mRNA recruits several host proteins such as up-frameshift suppressor 1 homolog (UPF1) and the DEAD (Asp-Glu-Ala-Asp) box 3- (DDX3) RNA helicase that will ensure an efficient association with the host translational machinery in order to synthesize the major structural proteins GAG and GAG–POL. In contrast to fully spliced transcripts, the unspliced mRNA does not undergo mRNA turnover as it is used as the viral genome incorporated into viral particles. CA, capsid protein; DCP, decapping enzyme; IN, integrase; MA, matrix protein; MOV10; Moloney leukemia virus 10; Myr, N-terminally myristoylated; NC, nucleocapsid protein; Pan2/3, PAB-dependent poly(A)-specific ribonuclease; p6, p6 protein; Ran-GTP, ras-related nuclear protein GTP; RT, reverse transcriptase; XRN1, 5'-3' Exoribonuclease 1.

HIV-1 is the prototype member of the *Lentivirus* genus of the *Retroviridae* family and the etiologic agent of the acquired immunodeficiency syndrome (AIDS). The HIV-1 genome consists of a 9 kb single-stranded RNA molecule carrying nine open reading frames that give rise to 15 viral proteins [16]. Moreover, transcription from the 3′-long terminal repeat (LTR) promoter gives rise to an additional protein named antisense protein (ASP), which seems to be important for HIV spread and pandemics [17]. Once integrated into a host chromosome, HIV-1 gene expression is regulated at the transcriptional and post-transcriptional levels by viral proteins TAT and REV, which are supported by several host proteins [18] (Figure 1). Host RNA polymerase II drives the synthesis of the full-length 9 kb mRNA, which is identical to the genomic RNA (gRNA) present within viral particles. Early during viral gene expression, the full-length transcript recruits the host mRNA processing machinery and undergoes alternative splicing, generating a subset of fully spliced (2 kb) and partially spliced (4 kb) transcripts, which in addition to the unspliced mRNA, are responsible for the synthesis of all viral proteins [19,20,21,22]. Fully spliced transcripts (used for the synthesis of TAT, REV, and NEF) follow the same classical gene expression pathway as cellular mRNAs, in which the rates of nuclear export and translation are highly stimulated by the splicing-dependent recruitment of nuclear proteins including the mRNA export factor NXF1 and the exon junction complex (EJC), amongst others (Figure 1). In sharp contrast, partially spliced transcripts (coding for ENV, VPU, VIF, and VPR) [19,20] and the unspliced mRNA (used for GAG and GAG–POL synthesis) [20] are not able to follow the classical mRNA nuclear export pathway due to the presence of introns and thus rely on an alternative mechanism of nuclear export in order to evade NXF1-associated quality controls.

## 3. The Full-Length and Partially Spliced mRNAs of HIV-1 Evade Nuclear Surveillance and Quality Control Cellular Mechanisms

As mentioned above, fully spliced transcripts follow the classical gene expression pathway employed by cellular mRNAs and are expected to undergo nucleoporin Tpr-mediated surveillance at the nuclear pore complex [23,24]. In sharp contrast, partially spliced transcripts and the unspliced mRNA are not able to follow the classical mRNA nuclear export pathway due to the presence of introns, which are recognized by this NXF1-associated mRNA surveillance mechanism, which induces nuclear retention and degradation of unprocessed transcripts [23,24]. However, the virus has evolved the REV protein, which binds to a specific RNA structure (the REV-responsive element or RRE) present exclusively within these intron-containing transcripts and to the host karyopherin chromosomal maintenance 1 (CRM1) [25,26,27]. In addition to the leucine-rich nuclear export signal (NES) that allows its association with CRM1, REV also possess a nuclear localization signal (NLS) that is recognized by importins-α/β, allowing for shuttling between the nucleus and cytoplasm through nuclear pore complexes (NPCs) [28,29,30].

As mentioned above, the REV protein was shown to be required for the transport of the unspliced and partially spliced mRNAs from the nucleus to the cytoplasm by a non-canonical mRNA export pathway [18,31]. Indeed, these intron-containing viral mRNAs are retained and degraded in the nucleus in the absence of REV because of their incomplete splicing [32]. At the molecular level, REV binds and oligomerizes along the 351nt RRE located within the *env* gene and thus is present exclusively in all underspliced viral transcripts [33,34]. Once synthesized, REV is imported and accumulates in the nucleus, where it binds to RRE-containing transcripts to promote their export via CRM1 [35]. It is noteworthy that the main function of CRM1 is related to the nuclear export of NES-containing proteins and small RNAs, being only infrequently involved in cellular mRNA export [35]. As a consequence, intron-containing viral transcripts avoid the mRNA surveillance and quality control mechanisms associated with the canonical nuclear export pathway and accumulate in the cytoplasm for translation [35] (Figure 1).

In addition to CRM1, a large number of cellular proteins have been shown to influence REV’s functions in the nuclear export of unspliced and partially spliced viral mRNAs [36]. These host factors include Matrin 3, host nuclear matrix protein (MATR3), an important host factor required to stabilize the viral RNA through its interaction with REV/RRE [37], REV-interacting protein (RIP)–REV/Rex activation domain-binding protein (RAB) [38,39], eukaryotic translation initiation factor 5A (eIF5A), Src-associated substrate in mitosis of 68 kDa (Sam68), and RNA helicases such as DEAD (Asp-Glu-Ala-Asp) box 3 (DDX3) [40], DDX1 [41], and RNA helicase A (RHA) [42], amongst others. Whether these proteins contribute to the stabilization of the viral nuclear export of mRNP or whether they determine the cytoplasmic fate of the viral mRNAs once exported (translation or packaging) requires further investigation.

## 4. REV Stabilizes RNA Instability Elements Present within the HIV-1 Unspliced mRNA

In contrast to cytoplasmic mRNA quality control pathways such as NMD, nuclear mRNA turnover is less understood. Interestingly, HIV-1 intron-containing mRNAs undergo nuclear downregulation as they are further spliced to completion or degraded in the absence of REV [32,43,44,45]. In the late 1980s, Pavlakis´s group designed an experimental setting aimed to identify inhibitory sequences present within the HIV-1 genome and to further study their function on viral gene expression [46,47,48]. They identified and characterized an inhibitory sequence in the HIV-1 *gag* gene that was named INS-1 [46]. They showed that the INS-1 element does not contain any functional splice site and acts in *cis* by lowering steady-state mRNA levels. Thus, INS-1 appeared to function at the level of mRNA stability [46]. The authors suggested that the inhibitory effect of INS-1 could be overcome by the REV–RRE interaction, demonstrating that this sequence present within the *gag* gene was important for REV-regulated viral gene expression [46]. Subsequently, the same group and others described more inhibitory sequences present in other genomic locations such as the *gag*/*pol* intersection (IN) [45], and within the *pol* (*cis*-repressive sequences or CRS) [49] and *env* [48] genes. These elements were shown to interfere with viral gene expression by impairing mRNA stability, nucleocytoplasmic transport, and cap-dependent translation initiation [46,50]. Interestingly, REV counteracted the defects exerted by these mRNA instability elements, allowing efficient viral gene expression [51].

Later on, Schneider and colleagues observed that most of the regions linked to instability (INS) contained high AU contents. Interestingly, while all REV-dependent mRNAs have unusually high AU contents, the AU content of fully spliced mRNA species is much lower [52]. Indeed, the AU contents within INS regions vary between 46% and 92% (with the average AU content in cellular mRNA being around 50%). However, it has been observed that particularly unstable cellular mRNAs such as c-myc, c-fos, c-myb, granulocyte-macrophage colony-stimulating factor, or mRNAs coding for cytokines share unusually high AU contents, which are involved in the instability and rapid degradation of these transcripts [53]. Interestingly, some of the viral INS elements contain the AUUUA pentanucleotide, which corresponds to a signal (AU-rich element or ARE) known to trigger an mRNA decay pathway known as ARE-mediated decay [53,54,55,56]. It is important to note that REV is unable to export underspliced mRNAs that do not contain a functional INS and hence it was proposed that these instability regions are an integral component of REV regulation [50]. Several mRNA-binding proteins, including polypyrimidine tract-binding protein 1 (PTB)/heterogeneous nuclear ribonucleoprotein I (hnRNP I) [57], heterogeneous nuclear ribonucleoprotein A1 (hnRNPA1) [58], and poly(A) binding protein cytoplasmic 1 (PABPC1) [47], were shown to specifically bind to such elements in vitro. It has been suggested that these INS-binding factors may avoid the recognition of the unspliced mRNA by the splicing machinery and promote their association with REV, thus enabling their export and expression. However, the precise molecular mechanisms by which INS and INS-binding factors acts on HIV-1 gene expression are still uncertain.

Subsequently, Zolotukhin and coworkers showed that the INS region present within the HIV-1 gag mRNA was bound by the heterodimeric transcription/splicing factor p54nrb/polypyrimidine tract-binding protein-associated splicing factor (PSF) [51]. By performing functional assays, the authors showed that PSF subunits act at the post-transcriptional level via INS in order to inhibit gag mRNA expression [51]. The authors proposed that p54nrb and PSF were host factors mediating INS function through a probably novel mRNA regulatory pathway regulating HIV-1 unspliced mRNA expression. However, a recent report showed that PSF was a positive component of the REV-mediated axis, whose contribution was to ensure a pool of underspliced mRNAs available for REV activity [37].

More recently, Valiente-Echeverría and colleagues reported the inhibitory effect of INS-1 on HIV-1 internal ribosome entry site (IRES)-mediated translation initiation. By using heterologous bicistronic mRNAs and both in vitro and cell-based assays, the authors showed that ectopic expression of REV and hnRNPA1 partially rescued the inhibition of INS-1 on translation [59].

It is clear that INS and other instability sequences are *cis* elements important for HIV-1 RNA and protein homeostasis and that the viral protein REV is involved in such regulation. However, it is still unclear what molecular mechanisms are in play and thus further investigation is needed to better understand this regulation.

## 5. HIV-1 Recruits Factors Involved in Cytoplasmic mRNA Decay Pathways

Cytoplasmic quality control pathways include NMD [60,61], NSD [62], and NGD [7], each of which depends on mRNA translation. The main function of these cytoplasmic mRNA decay pathways is to ensure the fidelity of gene expression [63,64]. As mentioned above, other pathways of cytoplasmic mRNA decay are conditionally used to regulate gene expression of specific mRNA targets containing specific *cis*-acting elements such as ARE-mediated decay [11], miRNA-mediated mRNA decay [65], or Staufen STAU-mediated mRNA decay (SMD) [61,63,66].

### 5.1. Nonsense-Mediated Decay

**Figure 2:**
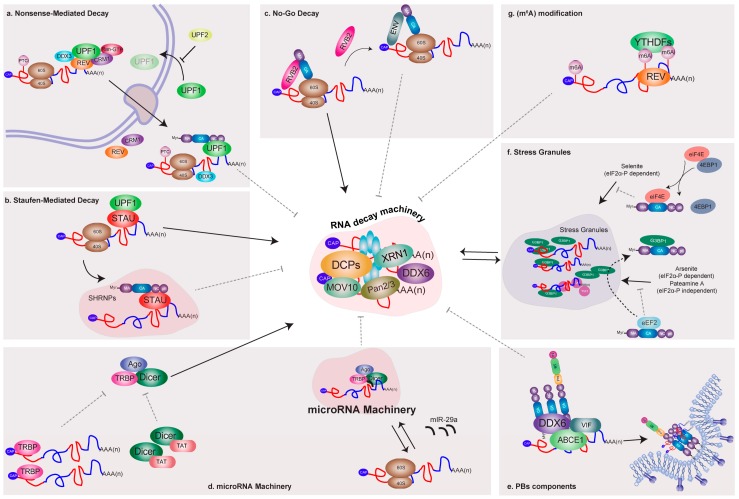
The HIV-1 unspliced mRNA has been shown to recruit components of different mRNA decay pathways including: (**a**) UPF1 (NMD, nonsense-mediated decay); (**b**) STAU1/2 (SMD, STAU-mediated mRNA decay); (**c**) RuvB-like 2 (RVB2) (NGD, No-Go decay); (**d**) HIV-1 *trans*-activating response (TAR) RNA-binding protein (TRBP) and Argonaute (Ago) (microRNA Machinery); (**e**) DDX6 (PBs, processing bodies); (**f**) eukaryotic elongation factor 2 (eEF2) and GTPase activating protein (GAP) SH3 domain-binding protein 1 (G3BP1) (SGs, stress granules); and (**g**) YTHDF2 (*N*^6^-methyladenosine (m^6^A)-dependent mRNA decay). Interestingly, most of these associations have been demonstrated to be beneficial for viral replication, suggesting that HIV-1 has evolved mechanisms to interact with these host factors in order to divert them from their functions in mRNA decay.

Nonsense-mediated mRNA decay is a quality control mechanism playing an important role in the degradation of mRNAs harboring premature termination codons (PTCs), thus avoiding the synthesis of truncated proteins that could be deleterious for the cell [67,68] (Figure 2). The activation of NMD depends on the conserved function of the UPF proteins UPF1, UPF2, and UPF3 [69,70]. UPF1 has a RNA helicase activity essential for NMD, while UPF2 serves as a bridge between UPF1 and UPF3. UPF3 interacts with the mRNA-bound EJC components eukaryotic translation initiation factor 4A3 (eIF4AIII), Y14, and Mago homolog (MAGOH) [71,72]. It has been estimated that around 5% to 20% of cellular mRNAs are NMD substrates, although it has not been established that every potential NMD substrate undergoes NMD-mediated degradation [71]. As viral mRNAs associate with the host machineries for processing, nuclear export, and translation, the question of how NMD affects viral mRNAs arises [72]. In this regard, various reports have shown that some RNA viruses have developed strategies to directly inhibit NMD and thus avoid this cytoplasmic mRNA degradation mechanism [73]. Several reports have shown that HIV-1 recruits the major NMD factor UPF1 to viral mRNPs containing the unspliced mRNA. In a pioneering report, Mouland´s group reported that UPF1 played unexpected roles in HIV-1 unspliced mRNA metabolism by promoting both nuclear export and translation [74]. In this work, the authors showed that UPF1 knockdown resulted in a strong decrease in HIV-1 unspliced mRNA levels and GAG expression (Figure 2a). Consistent with a positive effect on gene expression, the authors observed that overexpression of UPF1 resulted in enhanced levels of both the unspliced mRNA and GAG [74]. By using different mutants, the authors also demonstrated that the role of UPF1 in HIV-1 gene expression could be separated from its functions in NMD [74]. Interestingly, Hogg and Goff reported that UPF1 was able to sense reporter RNAs bearing the HIV-1 3′-untranslated region (UTR) and trigger mRNA decay in a 3′-UTR length-dependent manner [75]. However, further analyses using the whole virus confirmed that UPF1 indeed increases the levels of viral mRNA and the expression of GAG protein during the replication cycle [76,77]. Indeed, Mouland´s group provided further insights into the molecular mechanism by which UPF1 regulates the fate of the unspliced mRNA [76]. As such, Ajamian and colleagues demonstrated that UPF1 promotes HIV-1 unspliced mRNA gene expression by forming a specific complex with REV, CRM1, and the DEAD-box RNA helicase DDX3 [76] (Figure 2a). Interestingly, the authors also demonstrated that UPF2 was excluded from these specific UPF1/HIV-1 mRNP [76]. Protein–protein docking suggested that HIV-1 REV could bind UPF1 in a region that overlaps the UPF2 binding site. These in silico tests could explain the exclusion of UPF2, which acts as a negative regulator of gene expression from the unspliced mRNA [76]. The positive effects of UPF1 on HIV RNA metabolism reported in the context of a full replication cycle rather than with reporter RNAs support a model in which this host protein is not a decay-inducing factor for the HIV RNA [78].

Besides its functions on the post-transcriptional regulation of the unspliced mRNA, UPF1 was also shown to be critical for early events of the HIV-1 replication cycle. As such, Serquiña and coworkers reported that UPF1 knockdown or the ectopic expression of ATPase activity mutants resulted in reduced viral entry and reverse transcription (RT) [77]. Interestingly, the authors demonstrated that UPF1 was incorporated into viral particles through specific interactions with the nucleocapsid (NC) domain of GAG [77].

Interestingly, RNA editing enzymes such as the APOBEC3 cytidine deaminases may promote the generation of PTCs along the HIV RNA and thus its subsequent degradation through NMD. Indeed, in the absence of the viral protein Vif, apolipoprotein-B-mRNA-editing enzyme catalytic polypeptide-like 3G (APOBEC3G) generates as much as 20% of dC to dU changes by deamination of the minus-strand during the reverse transcription process [78,79]. However, it is still unknown whether APOBEC3-mediated hypermutations elicit NMD of the modified viral RNA.

Together, these results strongly indicate that the mRNA decay factor UPF1 is critical in determining the fate of the unspliced mRNA but also during the early steps of viral replication. The molecular determinants that interfere with the UPF1-mediated RNA decay pathway that senses the length of the HIV-1 3′-UTR in the context of a replication cycle are still unknown. Thus, further studies are required to determine whether UPF1 recruitment to the HIV-1 mRNPs interferes with RNA decay-promoting activities.

### 5.2. Staufen-Mediated mRNA Decay

Staufen (STAU) proteins are involved in multiple post-transcriptional regulatory processes, such as the regulation of mRNA transport and the activation of localized mRNA translation in neurons [80,81], as well as the binding to sequences present within the 3′-UTR of mRNAs [82,83]. Likewise, it has also been proposed that STAU can mediate the degradation of mRNA through the interaction with UPF1 in a process known as STAU-mediated mRNA decay [84].

STAU-mediated mRNA decay is an mRNA degradation process occurring in mammalian cells that is mediated by the binding of Staufen to a STAU1-binding site (SBS) present within the 3′- UTR of target mRNAs [84]. During this process, STAU1 recognizes dsRNA structures formed within the 3′-UTR of target mRNAs but also by an intermolecular association between the 3′-UTR of a target mRNA and complementary Alu elements present in long-noncoding RNA (lncRNA). The STAU1 paralog, STAU2, has also been reported to mediate SMD and both STAU proteins interact with UPF1, which is a key factor required for SMD [84]. Several reports have demonstrated that HIV-1 unspliced mRNA and GAG protein recruit STAU1 to form a specific viral mRNP (Figure 2b). By using a reporter gene harboring the *trans*-activating response region (TAR) at the 5′-end, Dugré-Brisson and colleagues presented evidence showing that STAU1 interacts with TAR, facilitating translation [85]. It was suggested that STAU1 might facilitate the nucleo–cytoplasmic transport of transcripts containing TAR and contribute to their interaction with the host translational machinery [85]. However, the mechanism by which this occurs has not yet been described. In addition, Chatel-Chaix and colleagues showed that STAU1 is an integral component of an intracellular HIV-1 ribonucleoprotein complex containing GAG [86,87,88]. Furthermore, the authors demonstrated that STAU1 interacts specifically with the NC domain of GAG in an RNA-independent manner [86]. The authors also showed that the HIV-1 unspliced mRNA co-immunoprecipitates together with STAU1, indicating that the viral mRNA is bound by STAU1 and the specific knockdown of STAU1 resulted in a significant reduction in viral infectivity [86]. Later, the same research group reported the assembly of a novel STAU1 RNP whose formation was dependent on HIV-1. STAU1, unspliced mRNA, and GAG colocalize in these STAU1 HIV-1-dependent RNPs (SHRNPs), the size of which depends on existing STAU1 levels in cells [89] (Figure 2b).

Other studies have determined that the human protein STAU2 promotes the export of HIV-1 mRNAs containing an RRE. This effect was shown to occur through the mRNA-independent interaction between REV and STAU2 [90]. Disruption of the REV–STAU2 interaction interferes with viral replication, indicating that recruitment of STAU2 to the RRE (which is located at the 3′-UTR of the unspliced mRNA) is critical for the HIV life cycle.

Together, these data strongly suggest that HIV-1 interacts with STAU proteins to form specific viral mRNPs that are required for efficient gene expression, trafficking, and viral particle assembly. It is unclear whether the recruitment of STAU proteins is related to a virally induced inhibition of SMD.

### 5.3. No-Go Decay

Recent findings suggest that HIV-1 may exploit the NGD pathway to fine-tune its own gene expression and ensure production of infectious virions. As such, Mu and colleagues showed that RuvB-like 2 (RVB2) inhibits HIV-1 GAG expression and that this inhibitory activity is antagonized by the viral ENV protein [91] (Figure 2c). These authors found that the HIV-1 unspliced mRNA is susceptible to NGD through a mechanism dependent on the translation of the matrix domain (MA) of GAG [91]. The authors also demonstrated that the RVB2 ATPase interacts with the HIV-1 5′-UTR and nascent MA peptides, impeding further translation of GAG or GAG–POL (Figure 2c). Thus, it was proposed that this mechanism mediated by RVB2 allows a balance between GAG and ENV by regulating the relative expression levels of these structural viral proteins necessary for efficient production of infectious viral particles [91]. Thus, by using the NGD mechanism, HIV-1 exploits a host RNA quality control pathway to maximize the quality of viral particles [91].

Together, these studies strongly suggest that HIV-1 proteins and/or RNA recruit factors involved in the translation-dependent degradation of cellular mRNAs such as UPF1 and STAU1 in order to ensure efficient viral replication. However, whether such interactions interfere with the mRNA degradation processes needs to be further investigated. Thus, studies aimed at identifying other factors that are involved in these pathways would be useful to clarify how the virus evades or interferes with mRNA quality control mechanisms.

## 6. Relationship between HIV and the Cellular microRNA Machinery and Processing Bodies (PBs) Components

RNA silencing is a mechanism for regulation of gene expression involving small non-coding RNA [92], as well as an innate host cell defense mechanism against viruses [93]. miRNA biogenesis begins with the RNA polymerase II-mediated transcription of miRNA precursor molecules containing a 5′-end cap structure and a 3′-end poly(A) tail. These long primary transcripts (pri-miRNAs) are cleaved by the Drosha–DGCR8 complex to produce 70 nt stem-loop structured precursors (pre-miRNAs), which are exported to the cytoplasm by exportin-5 and subsequently processed by Dicer [94]. Processing of the pre-miRNA by Dicer results in a mature miRNA guide strand that is loaded into the RNA-induced silencing complex (RISC) containing an Argonaute (Ago) protein and other RISC cofactors to form the microRNA-inducing silencing complex (miRISC) [94]. Mature miRISC targets specific mRNAs for translational repression or degradation [95]. Importantly, several components related to miRISC, such as miRNAs, mRNAs repressed by miRNAs, Ago proteins, DDX6, and Moloney leukemia virus 10 (MOV10), together with the mRNA degradation machinery, localize in PBs [96]. In fact, miRNA-mediated translational repression consistently correlates with mRNA accumulation in PBs [96].

One of the first works connecting HIV-1 with the cellular microRNA machinery was by Haase and colleagues, who described the identification of HIV-1 *trans*-activating response RNA-binding protein (TRBP) as a protein partner of human Dicer [97]. They showed that TRBP is required for optimal RNA silencing mediated by siRNAs and endogenous miRNAs, most probably by facilitating the cleavage of pre-miRNA [97] (Figure 2d). Then, Triboulet and colleagues demonstrated for the first time the physiological role of the miRNA-silencing machinery in controlling HIV-1 replication [98]. The authors showed that Type III RNAses Dicer and Drosha inhibited virus replication both in peripheral blood mononuclear cells from HIV-1-infected donors and in latently infected cells [98]. In turn, HIV-1 actively suppressed the expression of the polycistronic miRNA cluster miR-17/92, a miRNA cluster involved in genomic amplification in malignant lymphoma and lung cancer [99,100,101]. This specific suppression of the miR-17/92 cluster was found to be required for efficient viral replication and was dependent on p300/CBP-associated factor (PCAF), a histone acetyltransferase cofactor of TAT [98].

Subsequently, Nathans and colleagues reported that HIV-1 mRNA interacts with miRISC proteins and that disrupting PBs’ structures resulted in enhanced viral production and infectivity [95]. The authors found that HIV-1 mRNAs are susceptible to targeting by the human miRNA miR-29a, which induces the association of viral mRNAs with miRISC. The authors also showed that miR-29a represses viral replication by inducing an accumulation of viral mRNA in PBs [95] (Figure 2d).

Another protein component of PBs shown to be important for miRNA-mediated repression that has been involved with the HIV replication cycle is MOV10. This protein belongs to the UPF1-like subfamily of DExD-box RNA helicases and has ATP-dependent 5′ to 3′ directional RNA helicase activity [102,103]. MOV10 was co-purified with APOBEC3G/A3G and shown to affect the assembly and maturation of miRISC [104]. In 2010, Burdick and colleagues reported that MOV10 overexpression resulted in reduced levels of both GAG protein and virus production [105]. The authors showed that MOV10 was efficiently incorporated into virions, reducing virus infectivity, in part, by interfering with reverse transcription [105]. In addition, MOV10 overexpression reduced the proteolytic processing of GAG by the viral protease and the authors showed that MOV10 specifically associates with HIV-1 unspliced mRNA [105]. Curiously, these authors showed that knockdown of MOV10 decreased virus production but showed little impact on virus infectivity, suggesting that basal levels of MOV10 are required for efficient viral replication [105]. Consistent with this last observation, Huang and colleagues demonstrated that MOV10 potently enhances nuclear export of viral mRNAs through the REV–RRE axis and subsequently increases the expression of GAG protein and other late products [106]. The authors also observed that MOV10 interacts with REV in an RNA-independent manner [106]. Given the discrepancies between both reports, further research is necessary to elucidate the role of MOV10 protein during the HIV-1 replication cycle.

As mentioned above, PBs are cytoplasmic foci associated with the mRNA decay machinery as they contain mRNA decapping enzymes (Dcp1/2) and the 5′-3′ exonuclease XRN1 [107,108], deadenylation factors (Ccr1, Caf1, Not1) [109], NMD-associated proteins (SMG5-6-7, UPF1) [107,110], and translational repressors (CPEB, eIF4E-T, DDX6) [111,112]. Co-localization of miRISC and target mRNAs in PBs suggests that they function in miRNA-mediated gene silencing by sequestering target mRNA for storage or decay [113,114,115]. Indeed, several PB components such as GW182 and DDX6 (RCK/p54) play important roles in miRNA-dependent translational repression [116].

Besides the interactions of HIV-1 with the miRISC machinery described above, several reports have shown that HIV-1 co-opts some PBs components to promote viral replication. It has been reported that depletion of Ago2 or DDX6 produces inhibition of HIV-1 replication, indicating a role of these PBs-associated proteins in the viral life cycle [117,118]. Indeed, Reed and colleagues demonstrated that the assembly intermediates (AIs), containing HIV-1 GAG, GAG–POL, and VIF [119], are formed by the recruitment of DDX6 and ATP-binding cassette protein E1 (ABCE1), thus providing evidence that HIV-1 utilizes these factors to catalyze the assembly of immature capsid intermediates [120] (Figure 2e). Interestingly, Abrahamyan and colleagues showed a dramatic decrease of PBs around HIV-1 unspliced mRNA-containing foci, suggesting a local dissolution of PBs close to assembly sites [89].

## 7. Interactions of HIV-1 and Components of RNA Granules Involved in mRNA Silencing

In response to environmental stress such as viral infection, eukaryotic cells reprogram their translational machinery to allow the selective expression of proteins required for cell viability in the face of changing conditions. mRNAs encoding constitutively expressed proteins are redirected from polysomes to RNA granules during stress conditions. Two of these RNA granules have been well characterized in yeast and mammalian cells, SGs, which correspond to translationally silent sites of RNA storage, and PBs, which are foci involved in mRNA degradation [121]. During stress, SG assembly signaling can be triggered by the phosphorylation of translation initiation factor eIF2α, which reduces the availability of the eIF2–GTP–tRNA^Met^ ternary complex necessary during translation initiation [122,123]. Interestingly, HIV-1 replication was shown to interfere with SG assembly in favor of the assembly of viral specific mRNP containing STAU1 [89]. More recently, Valiente-Echevería and colleagues demonstrated that the HIV-1 GAG protein blocks SG assembly through an interaction between the N-terminal domain (NTD) of the capsid domain and the host eukaryotic elongation factor 2 (eEF2) [124] (Figure 2f). The authors also reported that GAG could mediate the disassembly of pre-existing SGs via an interaction with the SGs-dependency factor GTPase activating protein (GAP) SH3 domain-binding protein 1 (G3BP1) [124]. Interestingly, the mechanism by which HIV-1 interferes with SG assembly depends on the nature of the stressor. Indeed, the blockade of selenite-induced SGs was dependent on activation of eukaryotic translation initiation factor 4E binding protein 1 (4E-BP1) and the consequent inhibition of cap-binding by eIF4E [125] (Figure 2f). More recent data showed that G3BP1 binds the HIV-1 unspliced mRNA in the cytoplasm of macrophages to inhibit viral replication, supporting a role for G3BP1 and probably SGs as restriction factors that must be counteracted by HIV-1 [126].

## 8. Control of HIV-1 mRNA Abundance by Methylation of Viral Transcripts

Although hundreds of chemical modifications have been described in RNA, much less is known regarding the mechanisms and functions of these marks [127]. Methylation at the *N*^6^ position of adenosines is the most abundant internal modification identified in mRNAs and lncRNAs in many eukaryotic species, including yeast and mammals [128]. Accumulating evidence suggests that m^6^A regulates mRNA metabolism post-transcriptionally by altering the processing, nuclear export, translation, or stability of the modified mRNA [128].

It has been known for almost 40 years that, in addition to cellular mRNA, the RNAs of the influenza virus, adenovirus, Rous sarcoma virus (RSV), herpes simplex virus type 1 (HSV-1), and simian virus 40 (SV40) are m^6^A-modified [129,130,131,132,133]. Although the precise impact of this modification on viral replication still remains unclear, recent studies revealed that the presence of the m^6^A modification in the HIV-1 unspliced mRNA significantly affects gene expression and viral replication [134,135,136].

The post-transcriptional addition of m^6^A to mRNAs occurs predominantly in the nucleus and is catalyzed by a heterotrimeric protein complex consisting of two methyltransferase-like enzymes, METTL3 and METTL14, and the cofactor Wilms’ tumor 1-associated protein (WTAP), which together are recognized as the “writers” of m^6^A [137,138,139]. Fat mass and obesity-associated protein (FTO) and α-ketoglutarate-dependent dioxygenase homolog 5 (ALKBH5) are two specific m^6^A RNA demethylases (“erasers” of m^6^A) responsible for adenosine demethylation and its associated regulation [140,141]. The main “readers” of m^6^A on mRNAs are members of the so-called YTH domain-containing family. Amongst them, the cytoplasmic YTH proteins YTHDF1, YTHDF2, and YTHDF3, and the nuclear protein YTHDC1, have been shown to bind directly to m^6^A-containing mRNAs [13,14,139,142]. Recent studies indicated that m^6^A binding by YTHDF1 results in enhanced translational rates of its targets due to a specific interaction between YTHDF1 and eukaryotic initiation factor 3 (eIF3) [13]. In contrast, binding of m^6^A by YTHDF2 results in both the localization of its mRNA targets in PBs and concomitant accelerated degradation [143]. m^6^A-mediated mRNA degradation was shown to occur by the interaction between YTHDF2 and the CCR4–NOT deadenylase complex [128]. The precise function of YTHDF3 is still unclear [13,14,144]. Besides the mRNA degradation induced by binding of YTHDF2 to m^6^A, there is also evidence suggesting that the presence of this chemical modification could indirectly destabilize some transcripts by preventing the binding of the mRNA stabilizing protein human antigen R (HuR) [145]. Moreover, m^6^A regulates mRNA alternative splicing both directly through the recruitment of the m^6^A reader YTHDC1 and indirectly by altering RNA structures close to the binding sites of the splicing factor heterogeneous nuclear ribonucleoprotein C (C1/C2) (hnRNPC) [142,146].

Recently, Lichinchi and colleagues reported that the HIV-1 unspliced mRNAs (and probably other viral transcripts) contain multiple m^6^A modifications along their sequences [135]. Interestingly, the authors also observed that viral infection in a CD4+ T-cell line resulted in increased m^6^A levels in cellular poly(A) RNA [135]. They also showed that methylation of two conserved adenosines within the stem loop II region of the RRE was important for binding of REV, resulting in enhanced rates of nuclear export of the methylated viral mRNA, thus revealing the importance of the m^6^A modification for nuclear export of Rev-dependent viral mRNAs [135]. Moreover, Kennedy and colleagues found four clusters of m^6^A modifications in the 3′-UTR region of the HIV-1 unspliced mRNA that enhanced viral gene expression by recruiting the three cytoplasmic m^6^A “readers” proteins YTHDF1, 2, and 3 [134]. Notably, the authors observed that HIV-1 replication was dependent on the levels of YTHDF2 expression in infected T cells. As such, the virus presented enhanced GAG synthesis and viral particle production when YTHDF2 was overexpressed, while GAG protein and viral titers were reduced when the YTHDF2 gene was knocked out by the clustered regularly interspaced short palindromic repeats (CRISPR)/CRISPR-associated protein-9 nuclease (Cas9) system [134]. Contrary to the roles of YTHDF2 in mRNA degradation, the effects of YTHDF2 on HIV-1 replication were associated with enhanced viral mRNA abundance. Together, these data identified m^6^A and the resultant recruitment of REV or YTHDF proteins as major positive regulators of HIV-1 mRNA abundance in the cytoplasm (Figure 2g) [134]. It is noteworthy that, similar to what has been reported for mRNA decay factors UPF1 and STAU1, these data suggest that HIV-1 uses the mRNA decay-associated m^6^A reader YTHDF2 to promote viral replication.

More recently, Tirumuru and colleagues showed that proteins YTHDF1–3 recognize incoming m^6^A-modified HIV-1 RNA and inhibit reverse transcription during the early steps of infection in both cell lines and primary CD4+ T-cells. Consistent with this observation, knockdown of YTHDF1–3 in cells resulted in enhanced reverse transcription products [136]. However, the same authors showed that silencing m^6^A writers decreased HIV-1 GAG protein expression in virus-producer cells, while silencing m^6^A erasers increased GAG expression. The authors concluded that m^6^A plays a negative role during reverse transcription and a positive role later during viral gene expression [136].

Further research is necessary to elucidate the precise role of YTHDFs proteins, particularly YTHDF2, during the HIV-1 replicative cycle.

## 9. Conclusions and Future Perspectives

The HIV-1 unspliced mRNA plays critical roles during viral replication since it is used (i) as the precursor mRNA molecule undergoing alternative splicing in order to generate the remaining viral transcripts; (ii) as the mRNA template for GAG and GAG–POL synthesis; and (iii) as the genome packaged into newly assembled viral particles.

Interestingly, this 9 kb viral transcript possesses retained introns and AU-rich sequences—both signatures being incompatible with nuclear export and mRNA stability. In addition, the unspliced mRNA recruits different host factors including UPF1, STAU1/2, and the recently characterized m^6^A reader protein YTHDF2, all of them associated with mRNA degradation. Despite all these constraints, HIV-1 has evolved mechanisms that ensure the presence and stability of the unspliced mRNA in the cytoplasm of host cells. The viral protein REV appears as a key factor, not only allowing the exit of the unspliced RNA from the nucleus by an alternative pathway and overcoming surveillance mechanisms but also interfering with *cis*-acting instability RNA elements and coordinating the recruitment of some of these mRNA decay factors that instead play positive roles in viral gene expression and virus production. The relationship between HIV-1 mRNAs and the host mRNA decay machinery has historically been a very poorly explored field. Whether viral proteins or the infection per se interfere with NMD, SMD, mRNA decapping, or deadenylation has to our knowledge never been evaluated. Further studies on this unexplored topic will help us to better understand the RNA biology behind HIV-1 replication and will certainly contribute to the development of new and novel drugs aimed at counteracting viral production and avoiding viral resistance.
